# Can social capital play a role in contracting services of family doctors in China? Reflections based on an integrative review

**DOI:** 10.1186/s12875-021-01431-x

**Published:** 2021-06-21

**Authors:** Xinglong Xu, Henry Asante Antwi, Lulin Zhou, Tehzeeb Mustafa, Ama Boafo-Arthur

**Affiliations:** 1 School of Management, Jiangsu University, 301 Xuefu Road, Zhenjiang, Jiangsu P.R. China; 2 Center for Health and Public Policy Res, earch, Jiangsu University, 301 Xuefu Road, Zhenjiang, Jiangsu P.R. China; 3 Shanghai Normal University, 2151 Gongji Road, Pudongxin, Shanghai, P.R. China; 4 School of Continuing and Distance Education, University of Ghana, P. O BoxLG25, Legon, Accra, Ghana

**Keywords:** Family doctor, General practitioners, Integrative review, Social capital, Social network, General practice, Family physician

## Abstract

**Background:**

The family doctors’ contract service problem is not about government management alone, but an interaction of a complex social environment. Consequently, the effect of contracted services of family doctors not only depends on policy incentives but also needs to win the participation, acknowledgement, and confidence of community residents. The purpose of this integrative review is to examine whether there is any significant evidence that social capital in the form of social networking groups and other forms of social groups have any positive impact on the acceptance and the effectiveness of family doctors' contractual services.

**Method:**

Research on qualitative, quantitative and hybrid methods published in peer-reviewed journals on the social capital role in the process of contract service of family doctors were eligible for inclusion. In view of the increasing attention paid to the contract service effect of family doctors during this period, a 10-year time scale was selected to ensure full coverage of relevant literature in the same period. In total, 809 articles were determined in the database retrieval results which were downloaded and transferred to the Mendeley reference application software.

**Results:**

Twelve articles met the inclusion criteria for this integrative review and the quality of the included studies were assessed using the published criteria for the critical appraisal of quantitative and qualitative research methods. Majority of the articles assessed reported that there was evidence of a positive link between social support, especially a sense of belonging and the presence of regular family doctors. The influencing factors of patients' contract behavior of studies conducted in China were social interaction of social capital, acceptance of the first contact in the community, year of investigation, and exposure to the public.

**Conclusion:**

The study affirms previous studies that suggest that social resources have the propensity to improve relationship between patients and clients and between doctors and peers for the benefit of the patients and the stability of the overall healthcare system. Through the integration of various social resources family doctor systems accelerate the development of community construction. These social capital (social network groups) can guide residents to use family doctor services to maintain health. Social capital can also help residents have a regular and reliable family doctor.

## Background

China continues to make significant strides in her effort to reform its primary healthcare system to provide effective and efficient patient care services to its citizens. The primary healthcare system in China is divided into rural and urban components but the organizations are very similar [38]. Generally, healthcare is provided through a three-tiered system. The first tier is village clinics and medical centers often manned by the barefoot doctors and physician assistants due to the absence of highly qualified medical personnel. The second tier is made up of township and community health clinics that function primarily as out-patient clinics. These centers also serve as referral centers for village clinics and often attract fairly qualified medical professionals. The county hospitals with very qualified medical practitioners complete the third level of health service delivery in Chinese sub-urban regions. Similarly, the urban health service delivery starts with factories and neighborhood health stations. These are supervised by the district hospital while the most serious cases were handled by municipal hospitals and tertiary hospitals [20].

Nevertheless, China’s healthcare system in general and primary healthcare in particular still faces challenges in structural characteristics, incentives and policies, and quality of care, all of which diminish its preparedness to care for its large, aging population amidst the growing prevalence of chronic non-communicable diseases. With an ever-increasing healthcare demands, individuals started contracted their own family physicians hence the family doctor contract services. To that extent, China introduced the family doctor system to strengthen its primary healthcare delivery services but the family doctor system is plagued with several social problems that have rendered it less attractive to both doctors and patients. The family doctor system is assailed with community health resource constraints, unmotivated doctors, lack of social participation etc. [30].

For example, Binder [2], many Chinese are not aware of the benefits of family doctor contract services. The policy of government at the onset was to encourage more people to enroll with much investment on effective strict supervision and quality family doctor contract services [6]. Naturally, the confidence of residents eroded with persistent unsatisfied medical services [6]. Moreover, only few doctors are willing to accept family doctor services in China due to poor remuneration, excessive supervision and poor career progression [20, 30]. As explained in [30], effective family doctors services depends on both policy incentives and winning the participation, acknowledgement, and confidence of community residents [30].

According to Zhou, et al. [40] social capital can be an effective way to resolve some of the social challenges affecting effective family practice in China. The concept of social capital has evolved through series of scholarly research studies across the globe. Between 1700 and 1900, Adam Smith, Marx Rousseau, Tocqueville etc. established the basic tenets of social capital theory through the social exchange theory and the psychological construct theory. Hanifan [17] first used the term “Social Capital” to refer to the goodwill, mutual sympathy, fellowship, and social intercourse that exist among families and group of individuals and families. Jacobs [19] also defines social capital as a tool for strengthening urban vitality. Thus social capital is social network used in stimulating positive relationships. The modern concept of social capital is derived from the ground breaking works of Coleman [7] and Bourdieu [3].

According to Coleman [7] social capital are features of social organizations such as networks, norms, and social trust that facilitates coordination and cooperation for mutual benefits. Bourdieu [3] on the other hand explains social capital as the aggregate of the actual or potential resources which linked to possession of a durable network of more or less institutionalized relationships of mutual acquaintance and recognition. In the midst of the insecurities of family doctor contract services, both doctors and patients need social capital to maintain their trust in the system. Doctors need social support from supervisors and colleagues to openly communicate their grievances, reduce burnout, improve wellbeing and become more engaged in the practice. On the part of patients, Shang et al. [30] discloses, traditionally, family doctors have been seen as “people doctors”, rather than “disease doctors”. They are expected to form informal social relationships with their patients as part of both preventive and medical care services. They must bring patients to the doorstep of the medical services through warmth, trust, mutual help, shared value and coordination of communal healthcare tasks. Therefore, social capital will have an impact on the effect of contracted services of family doctors [20, 26, 30].

Since May 2016, the Chinese government has expansively promoted family doctor contracted services throughout the country and made it the main task of expanding the transformation of the clinical care and healthcare system in this new era [21] but effective family practice goes beyond setting up a system. It also involves, having sound personnel, effective incentive methods, smooth referral channels, and service-oriented residents' needs but these are poorly integrated in China’s family doctor system [34]. The after-effect of COVID-19 has increased the urgency for China to strengthen it family doctor contract services. China believes that family doctor contract service has a great influence on the ability of health service at the grass-roots level, and its effectiveness and accessibility are the keys to the realization of universal health coverage [13]. This review summarizes the qualitative and quantitative results from articles and the effective impact of social capital (social network groups) in the contracted services of family doctors and its applicability in China. This paper also reviews the influence of social capital on the form and effect of family doctor services. To further explore the mechanism and management of social capital in the contracted services of family doctors.

## Methods

This particular integrative review was steered by the updated version of Whittemore and Knafl's comprehensive methodology review outline. This is a description of the mythological approach to integrative review that was published in 2005. Since then the model has become the basic benchmark and the conceptual structure of most integrative review due to its robustness especially in the field of healthcare. The model combines both quantitative and qualitative study findings on targeted topics and provides a comprehensive understanding of the review issues. The framework includes five stages, such as identification of the research problem, articles retrieval, evaluation of the data retrieved, analysis of the retrieved data, and the presentation of the findings [25]. The PRISMA guidelines were consulted to augment the process of reporting the study’s findings in order to ensure robustness of inference. The extent to which each of these stages were applied to this study is highlighted in the next sections.

### Identification of the problem

The purpose of the study is to examine whether there is any significant evidence that social capital in the form of social networking groups and other forms of social groups have any positive impact on the acceptance and the effectiveness of family doctors' contractual services. It also seeks to recommend any practical learning suggestions that can be applied to healthcare management projects, such as the family doctor services policy development in China. The main purpose of this integrative review is to thoroughly identify, select, evaluate/examine critically, and synthesize articles on social capital which can positively affect the effect of family doctor's contract services.

### Literature Search

A strict inclusion and exclusion criteria was set in the search for qualifying articles. Firstly the paper should have been peer reviewed published, abstracted or indexed in a recognized database such as such as Medline, Web of Science, Science Direct and PubMed. Pre-print databases with articles under review in high impact factor journal were also consulted for current information that is in the review process. Secondly, the articles should have been published in English language. The studies also included quantitative research, qualitative research and mixed research studies in so far as the focus is on the role of social capital and family doctor contract service. The included articles must primarily focus on China or compare the case of China and other countries. This criterion notwithstanding, some studies that were not China based were included because they have significantly explored and advanced the frontiers of social capital in family medicine. They include studies Small et al., [32] that highlight the benefits of peer support networks established in conjunction with doctors to help needy doctors and patients without access important medical information.

Another inclusion criterion was that the field of medicine is a highly specialized area hence specialized professional groups abound. Thus journals or publications by professional groups within the healthcare sector that bothered on family practice were also consulted. The family doctor contract services in China do not have a long history but interest dates back to more than a decade. For this reason, studies spanning a ten year period up to 2019 were selected to ensure full coverage of relevant literature in the same period Table 1.

**Table 1:** Terms used for the literature search

Article Search Terms	
“family doctor” OR “family physician” OR “general practice” OR “general practitioner” OR “family practice” OR “social capital” OR “social network” OR “family doctor” AND “social capital” OR “primary care” AND “social capital” OR “family physician” AND “social capital” OR “service form for family doctor” OR “service results for family doctor”	

**Fig. 1:**
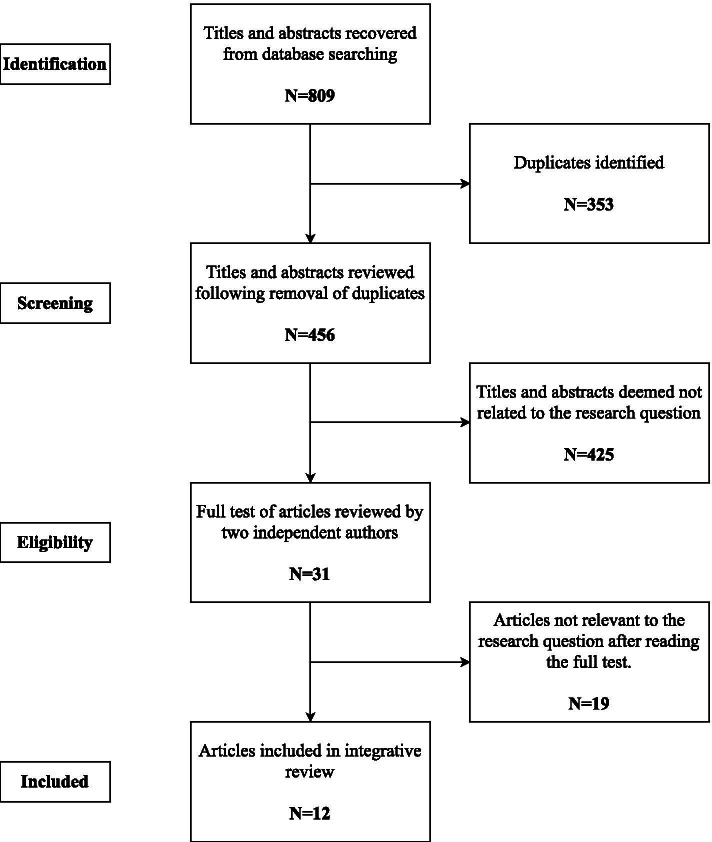
The prisma flow diagram

In total, 809 articles were determined in the database retrieval results which were downloaded and transferred to the Mendeley reference application software. Repeated articles (353) were deleted after which all the authors reviewed the copies of the papers and discussed their differences until a consensus was reached. After deliberation, 456 articles were further deleted. Article titles and their summaries were examined by XX and HAA for their importance based on the exclusion and inclusion criteria. After the screening, the two authors discussed their outcome with the other authors and 31 articles were selected for full-text assessment. On this basis, the authors further screened the above-mentioned articles around the research topic and objectives and forwarded 12 papers for analysis (Fig. 1).

### Data extraction and evaluation

The assessment of the 12 full-text articles composed of 2 level evaluations.

1. The first stage evaluation involved the exclusion of studies using the layout for the exclusion and inclusion criteria rules. In this level, 12 articles were selected based on the inclusion criteria. These comprise of 1 qualitative and 11 quantitative studies as indicated in (Fig. 1). Data were extracted on the study objectives, sampling strategy, sample size, study design, data collection technique, strengths and limitations, key findings, and analytical approach of the studies (Table 2).
2. The evaluation of the second level involved the critical appraisal (XX, ZLL, HAA, and JOM) to determine the methodological quality of the included studies. Due to the variety of methodologies and designs, two method-specific tools were identified to assess the quality of evidence. For qualitative studies, the Critical Appraisal Skills Programme (CASP) [9] tool was used (Table 3) and the Rees et. al. [28] survey checklist (Table 4) was utilized for cross-sectional studies. Each criterion was recorded as “Yes” or “No” or “Clear” or “Unclear” and results of appraisal were discussed between XX, ZLL, HAA and JOM with discrepancies resolved by consensus. Overall studies were found to be of good methodological quality with the only qualitative study recording nine out of the ten appraisal criteria (Table 3) and quantitative studies recording between 11 and 14 of the total appraisal score (Table 4). All the studies have determined the research objectives, proved the appropriateness of the design, used a clear sampling strategy, made a clear statement of the research results, and outlined the value of its research. In terms of the quantitative studies, response rates varied between 65.5% [26] to 97.75% [30] and only a study attempted to contact non-responders [26]. Most of the studies were limited to the purposive sampling method (Table 4). Four studies did not provide sufficient information to appraise the reliability of the measurement items (Table 4). Five of the quantitative studies were unclear regarding ethical approval or informed consent from an ethical committee (Table 4). Similarly, the conclusions of all quantitative studies are supported by data used for analysis, and the objectives of the study are described (Table 4). Qualitative research is valuable and provides details that fully consider the relationship between researchers and participants (Table 3).

**Table 2:** Descriptive characteristics of the studies included in the review

Title, Author, publication year and country	Study aim	Design	Sample strategy and sample size	Data collection method	Analytical approach	Strengths and /Limitations	Key findings reported by authors
Factors influencing patients’ contract choice with general practitioners in shanghai: a preliminary study [20] CHINA	To analyze the main influencing factors of contract behavior, including the concept of social capital, and put forward some suggestions for further development of the GP system of health care	A cross-sectional survey	A random sampling of 1200 patients	Questionnaire	Descriptive and t*-*test, χ^2^ test, factor analysis, and logistic regression analysis were used to analyze the data	The main limitation of this study was that just one district was studied in Shanghai	The influencing factors of patients' contract behavior were age (OR = 1.03; 95% CI = 1.02–1.04), education (OR = 0.83; 95% CI = 0.75–0.93), social interaction of social capital (OR = 1.34; 95% CI = 1.15–1.56), acceptance of first contact in community (OR = 3.25; 95% CI = 2.07–5.12), year of investigation (OR = 2.58; 95% CI = 1.92–3.47), and exposure to the public (OR = 1.60; 95% CI = 1.39–1.85)
Social capital and frequent attenders in general practice: a register-based cohort study [26] Denmark	To explore the association between frequent attendance and individual social capital	Based on a cohort study survey	The sampling for participants used a municipality stratified random sampling strategy and in total 35,700 residents, above the age of 16, were sampled from 579,000 inhabitants in 11 municipalities	The survey was distributed in a paper by mail in February 2010, and both paper and online responses were possible	Descriptive and multiple logistic regression analysis	This study was conducted explicitly at the individual level, whereas previous literature has featured multilevel or area-level analyses, which might influence the findings	Frequent attendance was associated with lower scores (adjusted for age, education, and income) in women's interpersonal trust (OR 0.86 (0.79 – 0.94)) and social networking (OR 0.88 (0.79 – 0.98)). There was no significant relationship between reciprocal norms and citizen participation and frequent attendance of women (1.05 (0.99–1.11) and 1.01 (0.92–1.11), respectively. These associations were not statistically significant for men
Social networks and the probability of having a regular family doctor [10] Canada	To examine the role played by social supports in helping to explain why a significant portion of the Canadian population does not have a regular family doctor even though primary care is fully covered by the public insurer and when having a regular physician is associated with better care and with access to specialists	Cross-Sectional	Five Canadian Community Health Surveys spanning 2001 to 2010 (*n* = 13,872 to *n* = 30,814)	Extraction of data from a community survey	estimated coefficients from a probit model	Continuing to develop ways of quantifying social supports and incorporating them into longitudinal surveys would help facilitate empirical analyses on how social supports affect health- care utilization	There was evidence that there is a positive link between social support, especially a sense of belonging and the presence of regular doctors
Social capital and having a regular family doctor: Evidence from longitudinal data, [1] Canada	to examine the impact of social capital (e.g., tangible support, friends, and family) on having a regular family doctor taking into account that social capital may be endogenously determined	longitudinal survey data	the Canadian National Population Health longitudinal survey (1994–2010: *n* = 41,022)	the Canadian National Population Health longitudinal survey (1994–2010)	dynamic random effects probit model	Since past access to a family doctor is a strong predictor of both current and future access, we show that social capital is much more important in helping individuals find a family doctor than for keeping one	There was evidence that there is a statistically significant positive causal relationship between social capital and the possibility of having regular family doctors
Social support, flexible resources, and health care navigation, [15] USA	To examine how social support operates as a flexible resource that helps people navigate the health care system	Qualitative study design	The study combines in-depth interviews with parents of pediatric cancer patients (*N* = 80), direct observation of clinical interactions between families and physicians (*N* = 73), and in-depth interviews with pediatric oncologists (*N* = 8)	Interview and observational methods	Descriptive and qualitative data analysis software NVivo 8	Results from this study improve understanding of the mechanisms that underlie the development and deployment of strategies for navigating the health care system and highlight the role of social support as a flexible resource that helps people meet institutional expectations for appropriate involvement in health care	The results show that doctors evaluate parents' visibility in hospital, medical vigilance and adherence to children's treatment, and use these judgments to form clinical decisions. Parents who get help from personal networks are more flexible in balancing competing needs, which enables parents to more effectively meet the agency's expectations of parents' proper participation in child care
Residents’ Awareness of Family Doctor Contract Services, Status of Contract with a Family Doctor, and Contract Service Needs in Zhejiang Province, China: A Cross-Sectional Study, [30] CHINA	The aim of this study was to investigate the residents’ awareness of Zhejiang Province, China, of family doctor contract services, the status of signing such a contract, and the demand for service items in the contracted service package	Cross-sectional Survey	enrolled 3960 residents from nine counties in Zhejiang Province using a multistage stratified random sampling method	self-designed questionnaire	Descriptive and analytical. Data were analyzed by SPSS 21.0	On the one hand, this was a cross-sectional study using a multistage stratified random sampling method to select respondents. As we know, most young people go out to work and the elderly stay at home, which may lead to under-representation of the whole population in Zhejiang Province	Health consultation (84.64%), regular physical examination (81.71%) and medical insurance reimbursement (80.06%) were the top three demands for contracted services. The awareness rate and signing rate of household doctors in Zhejiang Province are not ideal
General practitioners and carers: a questionnaire survey of attitudes, awareness of issues, barriers and enablers to the provision of services, [16] UK	to identify GPs’ attitudes, awareness of issues, and perceptions of the barriers and enablers to the provision of services	Cross-sectional Survey	Seventy-eight out of a total of 95 GPs	self-completion questionnaire	Descriptive and analytical	The study had a high response rate. A limitation is that the study participants had mostly chosen to attend a workshop on carers and may have been an atypical group motivated to support carers	General Practitioners consider time, resources and lack of knowledge as obstacles, but only 9% agreed that they can provide little support. However, 89% of the ten GPS (9) think they don't have enough training, and about half (47%) lack confidence
The influential role of personal advice networks on general practitioners’ performance: a social capital perspective, [5] Italy	The main goal of our study consists of assessing the influence of a GP’s social capital on her/his capacity to accomplish two organizational objectives related to his/her prescribing behaviour: containing health expenditures and fostering prescriptive standards	cross-sectional data from one LHA of the Italian NHS	Extracted data from the Italian National Health System, local health authorities (LHA) from 80 GPs’	A questionnaire was used for collecting data from archival sources of the Italian National Health System, local health authorities (LHA) from 80 GPs’	Descriptive and multivariate regression models	Alternatively, qualitative in-depth analyses could better explain the micro-dynamics behind the findings from this study	Social capital may affect the ability of a General Practitioner to achieve his/her goals. In particular, the higher the professional heterogeneity of the GP's personal counselling network, the lower his / her ability to achieve the specified appropriateness goals
Social relations and loneliness among older patients consulting their general practitioner, [12] Denmark	to analyze the social relations and loneliness of patients aged 65 years and above consulting their GP	Cross-sectional survey	Stratified sampling of Patients aged ≥ 65 years consulted their general practitioner in the study period in 12 practices in the Capital Region of Denmark (*N* = 459),	Questionnaire	Descriptive and analyzed using univariate logistic regression	it is a limitation that we only included patients who are able to visit the practice and to fill out the questionnaire. By not including those receiving home visits, very old and frail patients are probably underrepresented, and they are most likely lonelier than the respondents used in this study	36.2% of the people have a high degree of social participation, 45.5% have a medium degree of social participation, 18.3% have a low degree of social participation, 17.9% often or occasionally feel lonely. Higher social participation was associated with lower loneliness. Only 15.2% of autistic patients asked their GPs about their loneliness
Family physician–patient relationship and frequent attendance of primary and specialist health care: Results from a German population-based cohort study, [11] Germany	To investigate the association between the quality of the family physician–patient relationship and the frequent attendance of primary and specialist health care	Cohort study design	German population sample (*N* = 2266)	Patient-Doctor Relationship Questionnaire	Descriptive and multivariate analysis	Besides several strengths, like the use of a large sample representative of the German population and the use of a validated measure of perceived family physician–patient relationship quality, the study has some limitations. The study could not access health care use data of the participants available at health insurance companies	Frequent visits by family doctors were associated with lower income (OR 1.43, 95% CI 1.02–2.00), unpaid work (OR 1.58, CI 1.08–2.30), mental distress (OR 1.14, CI 1.07–1.22), physical symptoms (OR 1.07, CI 1.04–1.11), and comorbidity (OR 1.54, CI 1.36–1.74). Family doctors should be aware that demand factors, namely symptom burden and physical comorbidity, are the main drivers of frequent visits
No Spouse, No Son, No Daughter, No Kin in Contemporary China: Prevalence, Correlates, and Differences in Economic Support, [40] CHINA	To investigate the kin availability among adults aged 45 + in contemporary China, with an emphasis on child gender	Cross-sectional survey data extraction	nationally representative survey data from the China Health and Retirement Longitudinal Study (2011) With a multi-stage area probability sampling design	Extracted secondary data from a national survey	Descriptive and regression models analysis	N/A	In China, the proportion of people without relatives is very low (less than 2% of them do not have spouses/partners and children), but the availability of relatives is determined by gender, age group and socio-demographic characteristics. The proportion of the elderly without spouse/partner and daughter (3.2%) was more than twice that of the elderly without spouse/partner and son (1.4%). Adults without close relatives are disadvantaged in terms of health, wealth and financial support
Understanding the Association Between Perceived Financial Well‑Being and Life Satisfaction Among Older Adults: Does Social Capital Play a Role? [39] USA	The study examined the association between perceived financial well-being and life satisfaction while focusing on a potential mechanism, that is, whether or not social capital mediated the relationship between these two important factors in the lives of older adults	A longitudinal study of 2014 Health and Retirement Study (HRS)	4682 older adults (between the ages of 51 and 104) were included in the study sample	This study utilized data extracted from the 2014 Health and Retirement Study (HRS)	Descriptive and inferential analysis e.g. t-tests and F-tests were conducted and Ordinary Least Square (OLS) regression models were for the analysis of data	A study that will utilize more accurate variables that capture the bridging social capital would be desirable	Social capital plays a significant role in the relationship between financial well-being and life satisfaction of the elderly

**Table 3:** Methodological quality of qualitative studies

Study	1	2	3	4	5	6	7	8	9	10	Total Scores
E. Gage-Bouchard, [15]	Yes	Yes	Yes	Yes	Yes	Yes	Unclear	Yes	Yes	Clear	9/10

Keys: “1. Is there a clear statement of the purpose of the study?; 2. Whether the qualitative method is appropriate?; 3. Whether the research design is suitable for the research purpose?; 4. Whether the recruitment strategy is suitable for the purpose of research?; 5. Can data collection methods solve research problems?; 6. Whether the relationship between researchers and participants is fully considered?; 7. Whether moral issues are taken into consideration?; 8. Is data analysis rigorous enough?; 9. Whether there are clear findings?; 10. How valuable is this research?”

**Table 4:** Methodological quality of quantitative studies

Study	1a	2a	2b	2c	2d	3a	3b	3c	4a	4b	5a	6a	7a	8a	Total Scores
Jing et. al. [20]	Y	Y	Y	Y	N	Y	Y	Unclear	Y	Y	Y	Y	Y	Clear	12/14
Pasgaard et. al. [26]	Y	Y	Y	Y	Y	Y	Y	Y	Y	Y	Y	Y	Y	Clear	14/14
Devlin et. al. [10],	Y	Y	Y	N	N	Y	Y	Unclear	Y	Y	Y	Y	Y	Clear	11/14
Bataineh et. al. [1]	Y	Y	Y	N	N	Y	Y	Unclear	Y	Y	Y	Y	Y	Clear	11/14
X. Shang et. al. [30]	Y	Y	Y	Y	N	Y	Y	Y	Y	Y	Y	Y	Y	Clear	13/14
Greenwood et al. [16]	Y	Y	Y	Y	N	Y	Y	Y	Y	Y	Y	Y	Y	Clear	13/14
Calciolari et. al. [5]	Y	Y	Y	Y	N	Y	Y	Y	Y	Y	Y	Y	Y	Clear	13/14
Due, T. D., et. al. [12]	Y	Y	Y	N	N	Y	Y	Unclear	Y	Y	Y	Y	Y	Clear	11/14
A. Dinkel et. al. [11]	Y	Y	Y	Y	N	Y	Y	Y	Y	Y	Y	Y	Y	Clear	13/14
Z. Zhou et. al. [40]	Y	Y	Y	N	N	Y	Y	Y	Y	Y	Y	Y	Y	Clear	12/14
J. Yeo and Y. Lee, [39]	Y	Y	Y	N	N	Y	Y	Y	Y	Y	Y	Y	Y	Clear	12/14

Key:

Y – for Yes, N – for No. “A. Is the result valid? *1. Objectives:* 1a. Is the research objective clear? 2. *Design:* 2a. Whether the study design is suitable for the target? 2b. Does this theme represent all interested groups? 2c. Whether it has obtained moral/ethical recognition? 2d. Whether to take measures to contact non-responders? 3. Measurement and observation; 3a. Whether it is clear what has been measured, how to measure and what the result is? 3b. Is the measurement valid? 3c. Is the measurement result reliable? B What are the results; Presentation of results; 4a. Whether the basic data is fully described? 4b. Whether the results are clear, objective and detailed enough for the readers to make their own judgment? *Analysis;* 5a. Is the method used suitable for the collected data? C Will the results help locally? 6 *Discussion;* 6a Is the outcome of the discussion related to the existing knowledge about the discipline and research objectives? 7 *Interpretation*; 7a. Is the author's conclusion confirmed by data? 8 *Implementation;* 8a Can any necessary changes be implemented in practice?” Rees et al. [29]

### Retrieved data analysis

In light of the heterogeneity of the literature contained, the results of each study were examined [4], because conclusions can be drawn based on common factors [22]. Procedures which were used to carry out the thematic examination were guided by Smith et al. (V. [33], and Lucas *et. al.* [22].

### Findings presentation

The summary of the findings from the 12 studies included in the review is depicted in Table 2. The selected papers were mainly research works conducted in the USA [15, 39], Denmark [12, 26], Canada [1, 10], China [20, 30, 40], UK [16], Germany [11], and Italy [5]. Out of the eleven quantitative studies; seven of them were cross-sectional in nature [5, 10–12, 16, 30, 40]; two were based on data extracted from a cohort studies [11, 26] and the other two studies were based on longitudinal data [1, 39]. The sample size of these studies varied from 78 to 4682 respondents.

A study reported that the quality of social capital may invariably obstruct a General Practitioner’s propensity to achieve specific targets. Specifically, it is demonstrated that when a GP is networked in a highly heterogeneous social or professional group, the advice from this group can limit the extent to which he can make accurate personal prescription decisions [5].

Causes and affect relationships on family doctor contract services is also well established and discussed in the retrieved literature. Some studies confirmed the effect of social capital on the relationship between financial well-being and life satisfaction of the elderly [39]. Two studies retrieved from Canada also reported that there was evidence of a positive linkage between social support, especially a sense of belonging and the presence of regular family doctors [1, 10]. The low level of social participation affected family doctor services while higher social participation was associated with lower loneliness in studies conducted in Denmark [12, 26]. The effect of socio-economic on patient-family doctor behavior is also addressed by the studies. In the extant literature outside China, the main social factors that have been explored include education, income, gender and age. In the particular case of studies in China, the common social factors that moderates the social capital and doctor patient relationship were age, education, social interaction of social capital, acceptance of the first contact in the community, year of investigation, and exposure to the public [20, 30, 40].

Frequent attendance at GPs offices is a long standing critical issue for GPs because of its capacity to unduly impose excessive burden on the doctor. Moreover, frequent patient visits can be a huge drain on the limited healthcare resources. The interplay between social capital and frequent doctor visits came up in some of the reviewed studies especially in the context of Europe. One study sought to examine whether social capital resources can be harnessed to support or reduce frequent use of general practice, which may in turn lower the frequency to attend hospitals [26]. It emerged from this study that social capital deficit in some cases can induce frequent GP visits.

A German study linked the frequent visit of family doctors to their lower-income level [11]. The sample size of the qualitative study was 80 participants, including 59 women and 21 men [15]. This particular article reported on the examination of “how social support operated as a flexible resource that might help people navigate their health care system in their locality”. The study used interview and observational methods for data collection. The study also combined in-depth interviews and direct clinical interactions observations among families and their medical doctors. Descriptive and qualitative data were analyzed with the statistical software, NVivo version 8 [15]. The results from this study reported having improved the understanding of the mechanism that inspire the expansion and the distribution of the policies for steering the health care system. It further highlights the impact of social network support as a flexible resource that helped people met their organizational prospects for suitable involvement in health care [15].

To evaluate the quality of the studies, the Mixed Methods Appraisal Tool (MMAT) was applied as shown in Table 4. Pluye & Hong [27] explains that the MMAT tool helps to provide quality appraisal for quantitative, qualitative and mixed methods to be included in systematic reviews. In the MMAT the least paper met 11 out of the 14 quality benchmarks (3 articles) whereas the highest obtained 14 out of 14 quality benchmarks (1 article). This represents 25% and 8.3% MMAT quality respectively. While three other articles (also representing 25%) obtained met 12 of the 14 MMAT quality benchmark, the remaining 5 articles representing 41.6% obtained 13 out of the 14 MMAT quality benchmarks. The most frequent weaknesses related to lack of discussion on the reason for studying specific organisations, the influence of the organisation on the research and researcher influence in qualitative and mixed methods studies. There were also issues with lack of a clear description of the sampling process of respondents adopted by authors in quantitative studies and sub threshold rates for acceptable response or follow-up in non-randomized quantitative studies were also recorded as major weaknesses of the quantitative research. Most of the studies had support from funding agencies or organisations for whom the research outcome serve their interest. Thus the influence of such organisations in the conduct of the research was not disclosed by the researchers.

## Results

### Identification of social capital/network influence of family doctor acceptance

The theme identification of social capital /network influence of family doctor acceptance explores.

1. Benefits of having a family doctor
2. Benefits of belonging to social capital or social network
3. Influence of social capital or social network groups on accepting a family doctor
4. Family doctor policies
5. Barriers to the family doctor contract services acceptance

#### Benefits of having a family doctor

GPs play a unique role in dealing with social relations and loneliness [12]. GPs identified the important influence of general practice in supporting patients but also wished to get more training and support [16]. The family doctor is the first call point in an emergency situation. There is evidence that finding a regular family doctor can improve health [1]. Better continuity and quality of care, as well as improved health, are some of the overall benefits of having a regular family doctor [1].

#### Benefits of belonging to social capital or social network group

Undoubtedly, in terms of quality of life, morbidity, and mortality, the community social network relationships are very vital for the people in the community. This applies especially to the elderly [12]. The health of the people in the community, as well as their social network groups, are interconnected. In recognition of this social fact, over the past decade, there has been increasing conceptual and empirical attention to the impact of social networks on health [31]. According to a study conducted by E. A. Gage-Bouchard [15], most parents who received aids through their personal social networks were most comfortable in balancing their competing demands. This enabled them to effectively comply with their agencies’ hopes of parents' proper participation in child care [15]. In this way, social support provided some families with flexible resources, which enabled them to acclimatized to the needs of caring for children with cancer more quickly. It also raised a fruitful social relationship between parents and their medical care providers which played an active impact in the health of their children [15]. Notably, quite a number of studies on social support and health have shown that emotional, logistical, information and financial support from personal networks enhance people's coping choices in managing serious diseases [31, 35].

#### Influence of social capital or social network groups on accepting a family doctor

Some aspects of women's social capital groups are related to regular participation in the acceptance and use of GPs or family doctors [26]. On the contrary, men's social capital has nothing to do with their acceptance of family doctors or GPs. This shows that there are various and varied relationships between social capital and the gender of regular attendance [26]. In Zhejiang Province, China, for instance, the level of family doctor contract services (FDCS) awareness and the signing rate of family doctors are not the ideal, aside from that there is still a lot to be done for further improvement [30]. However, age, educational level and chronic medical history were some of the factors identified to be influencing residents' awareness of FDCS. Concurrently, residents' understanding of FDCS also affects their signing rate with family doctors. Surprisingly, patients who signed the agreement with the family doctors had a large request for FDCS. But the demand rate of residents with different social and demographic characteristics was different for different FDCS projects. Therefore, this is a call for the government to strengthen the policy support, strengthen the information campaign, expand the service scope, and provide more attractive service items in order to encourage the expansion of FDCS in China [30]. Residents should be allowed to choose a family doctor of their choice to sign the contract with. In addition, they should be allowed to also choose the projects they need when signing a contract with family doctors. The family doctors should also offer better services to the satisfaction and fulfilment of the residents [30].

There is evidence that a positive link exists between social support (especially a sense of belonging) and maintaining a consistent medical doctor [10]. There is also evidence that shows a significant positive causal association between social capital and the likelihood of attaining a regular family doctor [1]. Nevertheless, social capital was reported by a study as being much more important to helping individuals to find family doctors than to keeping them [1]. Undoubtedly, the influence of social capital (social networking groups) in the relationship between economic well-being and the life satisfaction of the elderly was statistically significant [39]. It is worth noting that obtaining social capital through strong family relationships and active social networks may decide the life satisfaction of the elderly [39].

#### Family doctor policies in China

Under the dual system of government guidance and market regulation, FDCS improves the quality of medical services through policy guidance and individual independent contracting [30]. Based on the government-led contract to provide a certain limit of service content, standardize service pricing, maintain service order, and adjust the exclusive personalized service according to the market demand. This system not only produces a unique medical service model but also faces a huge Governance Dilemma of doctor-patient trust and risk resolution. In order to give social power, give full play to the effective role of social organizations, build an interactive, integrated and trusted network governance structure, and straighten out the role relationship between the government and the market, it has an important role in promoting the integration of market and social resources across borders and regions and promoting the social empowerment of the government. Therefore, this policy is considered to be an effective way to promote the development of family doctor relationship under contracted services.

#### Barriers to the FDCS acceptance

Based on self completed questionnaire, Greenwood et al., [16] investigated the attitude of GPs to carers, their awareness and knowledge of issues affecting these carers as well as the barriers that hinder the effective functioning of their supporting carers. The GPs in this study indicated that the lack of time, resources and knowledge of inpatients or patients were some of the obstacles for patients or carers to accept the FDCS [16]. These GPs also recognize that they also have a vital role to play in supporting carers [16]. The promotion and coverage of FDCSs will be expanded, and personalized contract programs will be launched to meet the needs of different social network groups, so as to promote the rapid development of family doctor's contracts in all provinces of China [30]. The factors that affect residents' trust and satisfaction are family doctors' medical service skills, residents' familiarity with family doctors, communication ability of family doctors, patients' medical care concept and medical environment of community hospitals [8]. The other factors that influence the work attitude and activities of family doctors' services are work task and income level, the management of community health centre, and the understanding of their own occupation and service objective attitude [8]. The family doctor system should take the community as the carrier, integrate various social resources, improve the doctor-patient relationship, cultivate social network groups in the community, and accelerate the development of community construction. In addition to the support of hardware facilities and supporting policies, social capital, and other soft environments are of great significance to the establishment of FDCS [8]. 

## Discussion

The main aim of this integrative review report is to determine if the benefits of social networking groups tend to help or enable residents to better understand the family doctors' policies and make them healthy by using FDCS. It is of no doubt that a solid family doctor-patient relation doesn't certainly prevent frequent visits to a doctor’s office or to make appointments with a specialist. It does not also imply that GPs should not be concerned in building robust relationships with patients, as strong family doctor-patient relationships are related to other essential issues such as patients’ compliance and satisfaction [11, 14]. Over the past 20 years, the issues of social capital have been linked to a diversity of healthcare outcomes such as causes of mortality [18, 24]. Social capital(interpersonal relationships) is a complicated imaginary structure with a very intricate pedigree [23]. In fact, the understanding of social capital can be seen as the actual or likely benefits that individuals can obtain through their social network, such as nursing, advice, emotional and financial support. Therefore, in terms of health care exploitation, we expect closer ties and greater impact on their behaviour. Due to the fact that health is often discussed with family members and other close confidants, leading to the use of informal resources, which may reduce the desire for formal healthcare system. However, the relationship between social behaviour and health care utilization is complex. Frequent attendance is defined as a disproportionate amount of general practice consultation compared to the general public [36]. The evidence presented from various papers reviewed illustrates numerous ways in which personal health and good life affect the state of health and wellness of others [31]. The studies on the impact of social networks on health, the role of social support in determining individual health, and the spread of disease from one person to another have demonstrated the interconnectedness or interdependence of health among individuals in the society. In short, a person's illness, healthiness behaviour, infirmity, use of medical care facility, and death are related to similar outcomes in many other people that the person is associated with, and may have an abiotic spread of the disease. In the area of clinical and public health, the existence of social network health effects provides a strong theoretical and practical basis for the utilization and the healthiness of the people in the community. If a person's health outcome depends not only on his / her own biology and behaviour but also on the biology and behaviour of people around him/her, then collective intervention rather than individual intervention is particularly prominent. Social network groups exist to signify that individuals and events are interdependent, and health and healthcare can transcend individuals in ways that patients, doctors, decision-makers and researchers care about. The Chinese government vigorously promotes the family doctor policy system which requires everyone to have a family doctor especially for children and the elderly. The National Health and Family Planning Commission even has a system of assigning family doctors to families, yet many citizens are either ignorant of this requirement or have deliberately ignored it. Moreover, systemic constraints underpins why some residents do not have a registered family doctor. Firstly, the number of family doctors is not enough. The family doctors in China currently comprise of community GPs, retired doctors, and rural doctors. Since they also work as GPs, they do not have enough time and energy to work as family doctors with its peculiar demands. On the part of residents a common reason for low patronage is that many of them do not know the benefits the family doctor system can bring to them. Moreover sustained economic prosperity in China since its opening up in 1979, has led to richer citizens who have the means to afford high quality and exclusive care in big hospitals both at home and abroad.

## Conclusion

The study affirms previous studies that suggest that social resources has the propensity to improve relationship between patients and clients and between doctors and peers for the benefit of the patients and the stability of the overall healthcare system. This underscores the need for healthcare managers to harness, nurture and sustain the different social network systems within the organization to support effective health service delivery. With diligence these social networks can be transformed into a multitude of social capital resources that can be optimized and deployed to support a robust, reliable, responsive, effective and efficient family care delivery across the healthcare delivery strata.

With China’s strong collectivist culture, social capital is that it can help the Government to better promote and disseminate family doctor policies through the already established social network groups. Therefore understanding social capital or social network groups on the concept of family doctor system can promote and encourage residents to fully register and use family doctor services. The results of this review provide important rudimentary information for more advanced studies in the area of family doctor contract services in China and beyond. Firstly, while these study focuses on the extent to which social capital can play a role in patients desire to obtain family contract service, an emerging strand of literature equally suggest the reluctance of doctors to opt for family practice in China due to several factors. Some family doctors believe that an enormous demand is imposed by a bureaucracy that has little or no knowledge about the medical practice and it is not possible to protest. For example, the requirement for monitoring and evaluation of family doctors are perceived to be unfriendly and unsuitable to the healthcare industry even though they have been successfully applied in other industries. The need to stimulate practitioners to accept family doctor contract services is equally critical for the success of the venture and more systematic research is needed to advance the frontiers of knowledge in this regard.

Typical of academic studies a number of limitations may affect the findings of this research. Particularly this study focuses primarily on the interplay between social capital and family doctor contract services in China. This restriction severely limited the number of papers included (12) but also the scope regarding the potential of social capital. We recommend future research to consider widen the scope of research and explore other emerging benefits of social capital in family practice. For example, the rapid evolution of peer support networks established in conjunction with doctors is believed to be helping needy patients must be interrogated in a future studies.

Similarly, the studies were taken from only a few databases and supplemented with three additional sources. This implies that all other studies outside these sources were ignored. The small sample size of articles studied may limit the findings of this research. Relatedly, the strict inclusive and exclusive criteria used to select articles means that other articles with potentially useful information were deemed lower-quality, downgraded and disregarded. Further, the methodological limitations of the parent studies (particularly, regarding the sampling strategies of reviewed materials in the case of primary studies) limits the findings of the research. This is because most of these studies did not clearly indicate how participants in the studies were recruited and sampled and that may limit the transferability of the findings of this research. Even in the case of the secondary research based studies, the authors themselves have disclosed limitations regarding the process of sampling the studies which further limits any analysis made from them. This study included only articles published in English language and the coverage of the final set of admitted articles did not equally cover all the geographical areas of the world. This limits the generalizability of the findings to other contexts.

## Data Availability

The data for this research is held by the authors and will be made available upon reasonable request.

## Abbreviations

GPs: General practitioners
CASP: Critical appraisal skills programme
FDCS: Family doctor contract service
